# Impact of Molecular Dynamics of Polyrotaxanes on Chondrocytes in Double-Network Supramolecular Hydrogels under Physiological Thermomechanical Stimulation

**DOI:** 10.1021/acs.biomac.3c01132

**Published:** 2024-01-02

**Authors:** Theofanis Stampoultzis, Vijay Kumar Rana, Yanheng Guo, Dominique P. Pioletti

**Affiliations:** Laboratory of Biomechanical Orthopedics, Institute of Bioengineering, EPFL, Lausanne 1015, Switzerland

## Abstract

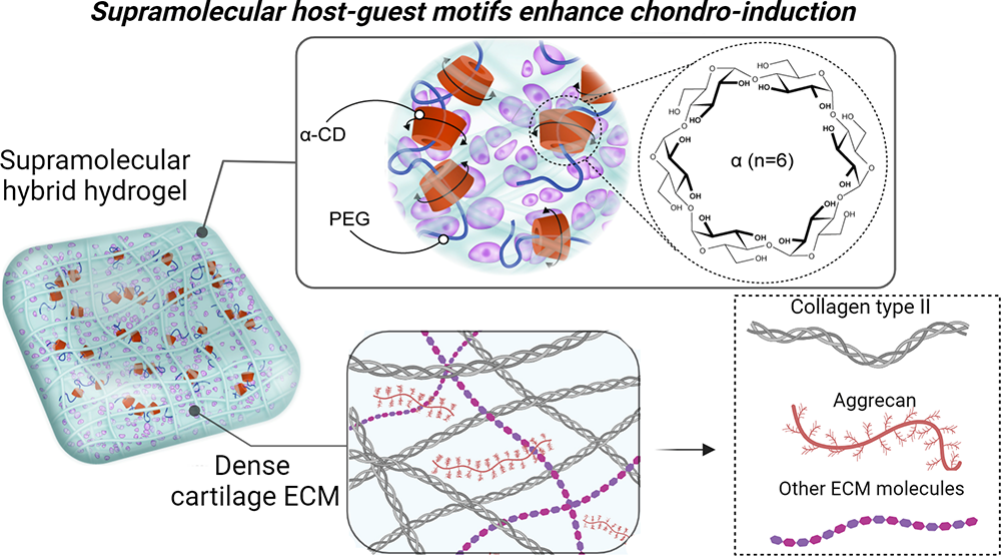

Hyaline cartilage, a soft tissue enriched with a dynamic extracellular matrix, manifests as a supramolecular system within load-bearing joints. At the same time, the challenge of cartilage repair through tissue engineering lies in replicating intricate cellular–matrix interactions. This study attempts to investigate chondrocyte responses within double-network supramolecular hybrid hydrogels tailored to mimic the dynamic molecular nature of hyaline cartilage. To this end, we infused noncovalent host–guest polyrotaxanes, by blending α-cyclodextrins as host molecules and polyethylene glycol as guests, into a gelatin-based covalent matrix, thereby enhancing its dynamic characteristics. Subsequently, chondrocytes were seeded into these hydrogels to systematically probe the effects of two concentrations of the introduced polyrotaxanes (instilling different levels of supramolecular dynamism in the hydrogel systems) on the cellular responsiveness. Our findings unveiled an augmented level of cellular mechanosensitivity for supramolecular hydrogels compared to pure covalent-based systems. This is demonstrated by an increased mRNA expression of ion channels (TREK1, TRPV4, and PIEZO1), signaling molecules (SOX9) and matrix-remodeling enzymes (LOXL2). Such outcomes were further elevated upon external application of biomimetic thermomechanical loading, which brought a stark increase in the accumulation of sulfated glycosaminoglycans and collagen. Overall, we found that matrix adaptability plays a pivotal role in modulating chondrocyte responses within double-network supramolecular hydrogels. These findings hold the potential for advancing cartilage engineering within load-bearing joints.

## 1 Introduction

In articular cartilage, chondrocytes and their dynamic local microenvironment constantly interact and communicate through biophysical and biochemical cues to regulate and guide various cell behaviors, such as cell differentiation.^1^ It is now globally accepted that dynamic temporal interactions predominantly mediated by chondrocyte adhesion to the extracellular matrix and applied biomechanical stimuli present a crucial role in transferring forces to and between cells that ultimately control chondrocyte function and tissue homeostasis.^2^ It is assumed that such interactions can be leveraged to alter cartilage disease and directly promote regeneration. Investigating the mechanisms by which physical cues and the nature of the cellular microenvironment are sensed by chondrocytes and how these are converted into biochemical signals is believed to be the gatekeeper to understand cartilage mechanobiology.^3^

Hydrogels are deemed highly attractive candidate materials to study how physical cues affect the chondrocyte responses in vitro, due to their inherent simplicity in terms of starting constituents and preparation, allowing for the precise control of their chemical and physical properties.^4^ Nonetheless, thus far, previous studies have primarily focused on utilizing hydrogels cross-linked through covalent bonds to enhance compression resistance in the case of applications for articulating joints. Despite their merits, covalent bonds tend to confine the synthesis of the extracellular matrix predominantly within the pericellular space.^5^ Conversely, biological tissues predominantly embrace the dominance of noncovalent interactions, presenting a stark divergence from the primarily covalent cross-linking strategies often observed in hydrogel investigations.^6^ In this regard, there is a growing interest in cell-adaptable hydrogels that can adjust and reorganize in response to mechanical stress or strain. As one example, supramolecular host–guest hydrogels are well-suited to study cartilage mechanobiology where the reversibility of the cross-links occurs under physiological conditions.^7^ Supramolecular chemistry is entrenched in the rational design of specific and reversible molecular recognition motifs capitalizing on dynamic noncovalent interactions to create organized systems.^8^ Because of the dynamic nature of noncovalent interactions, supramolecular materials can rapidly respond to multifarious external stimuli, thereby recreating aspects of the dynamics present in living systems, making them a suitable candidate for cartilage studies.^9^

Although covalent hydrogels can be engineered to possess elastic and/or viscoelastic mechanical properties,^10^ they fail to replicate the inherent dynamics of the extracellular matrix found in tissues like hyaline cartilage. Herein, we have devised an approach that combines a covalent-based hydrogel with supramolecular polyrotaxane motifs, aiming to capitalize on the beneficial properties of supramolecular host–guest interactions (dynamic reversibility) embedded within the covalent network. Polyrotaxanes are molecular assemblies that resemble beaded chains at the molecular scale.^11^ Typically, the chain component is constructed from long-chain polymers such as poly(ethylene glycol) (PEG), serving as the axle for the molecular assembly. This axle component provides a platform on which the ring molecules (e.g., cyclodextrins, CDs) can undergo various motions.^12^ PRXs exhibit controlled molecular mobility governed by factors such as CD-PEG ratios and intermolecular forces. This controlled mobility enables PRXs to emulate the noncovalent interactions/characteristics of soft tissues, suggesting their potential in biomaterials for mimicking such behaviors.^12^ This integration enables the creation of a more permissive environment for encapsulated cells to interact with the hydrogel matrix during externally applied deformation while simultaneously upholding the desirable characteristics, such as robustness, inherent in covalent bonding. By incorporating supramolecular polyrotaxanes, we enable the hydrogel to undergo reorganization and adaptability, mimicking the natural behavior of the extracellular matrix in the hyaline cartilage.

So far, despite its significance, a link between the temporal hierarchy of gel dynamics and adult chondrocyte behavior, particularly in response to externally applied biomimetic stimulation, remains elusive. Understanding how mechanobiological signals affect the chondrocyte behavior is crucial for enhancing the outcomes of tissue engineering approaches.^13^ Thus, we further sought to examine mechanobiological interactions among matrix characteristics upon an externally applied biomimetic thermomechanical load using host–guest supramolecular hydrogels. To the best of our knowledge, this research work represents the first study to explore interactive effects between dynamic reversible cross-links (polyrotaxanes) and physiologically relevant biomimetic thermomechanical loading for cartilage tissue engineering.

To this end, primary human chondrocytes encapsulated in supramolecular hydrogels were maintained in free swelling condition (static) as well as subjected to a long-term (up to 21 days) culture via a custom-made bioreactor apparatus designed to simulate transient thermomechanical stimuli as experienced in knee joints.^14^ PCR analysis was employed to investigate the early transcriptional interactions among hydrogels with static and dynamic host–guest cross-links (polyrotaxanes), revealing significant transcriptional changes between the experimental groups in a free swelling condition. Rates of biosynthesis were also analyzed by quantifying the deposition of sulfated glycosaminoglycans (sGAGs) and the total collagen type following thermomechanical stimulation. Histological analysis was further utilized to visualize and detect the spatial distributions of these molecules. Overall, this study underscores the significance of a dynamic extracellular matrix (ECM) akin to that found in native cartilage, accentuating how mechanobiological cues intricately guide chondrocyte biosynthetic responses within the dynamic hydrogel milieu.

## 2 Materials and Methods

α-Cyclodextrin was purchased from Sigma-Aldrich (C4642, ≥ 98%). Polyethylene glycol 2000 was purchased from Sigma-Aldrich (*M*_n_ = 2000 Da for synthesis). Gelatin type A from porcine skin (ref G2500) and methacrylic anhydride (ref 276685) were purchased from Sigma-Aldrich.

### 2.1 Preparation of Host–Guest Molecules and Supramolecular Hydrogels

The preparation of PEG/α-CD polyrotaxanes involved dissolving two different concentrations of α-cyclodextrin (α-CD) (12 and 36 mg) in 400 μL of PBS. This solution was then added to a 600 μL PEG/PBS solution (6.5 wt %). The mixture was thoroughly mixed and allowed to equilibrate (1 day prior to experiments) to ensure the formation of host–guest complexes. Methacrylated gelatin (GelMA) was synthesized as previously described^15^ and then dissolved in the [PEG-α-CD]/PBS system at a final concentration of 7 wt %. The solution was gently stirred and heated at 37 °C until the complete dissolution of GelMA was achieved. For the preparation of nonsupramolecular hydrogels, GelMA was dissolved in PBS at a final concentration of 7 wt %.

### 2.2 NMR Analysis and Isothermal Titration Calorimetry (ITC)

^1^H NMR spectra were acquired using a Bruker Avance NMR spectrometer (400 MHz) with a BBI probe and processed with MestReNOVA software, as previously described.^15^ Chemical shifts were reported in parts per million, rounded to the nearest 0.01 ppm for ^1^H NMR.

Isothermal titration calorimetry (ITC) experiments were conducted using a MicroCal PEAQ-ITC instrument (MicroCal Inc.) in pH 7 phosphate-buffered saline (PBS). The titration involved stepwise injections of α-CD solution (9 mg/mL) from a 250 μL injection syringe into a sample cell containing 0.6 wt % PEG. Each titration comprised 20 successive injections of α-CD solution into the reaction cell, which contained 1.5 mL of a PEG solution (1.316 mg/mL). Time intervals of 150 s were employed to ensure signal stabilization between injections. The first injection was set to a very small volume of 0.4 μL (due to the possible dilution during the equilibration time preceding the measurement, and then the first injection was ignored in the analysis of data) and was followed by 19 injections of 2 μL each. Continuous rotation of the syringe assembly at 750 rpm facilitated mixing. Both cells were in an adiabatic chamber at a constant temperature of 298.15 K. The ITC thermogram, depicting peaks corresponding to individual α-CD solution aliquots, illustrated the exothermic heat associated with the formation of polyrotaxane complex formation. Thermodynamic parameters were determined using the MicroCal software through nonlinear least-squares fitting to a standard single-site binding model, providing association constant (Ka), stoichiometry (N), and change in enthalpy (Δ*H*).

### 2.3 Experimental Groups

This study encompassed three distinct phases. In Phase I, the objective was to assess the effects of incorporating dynamic host–guest polyrotaxanes into a covalent network and their subsequent impact on chondroinduction, particularly on the expression of key chondrogenic genes. This evaluation involved a comparative analysis with motif-absent (single network) hydrogels under free-swelling conditions. Advancing to Phase II, the focus shifted to the identification of potential enhancements observed in Phase I, now at the protein level. Subsequently, Phase III involved subjecting the hydrogels to biomimetic thermomechanical stimulation under hypoxia as previously described.^16^ The overarching goal was to enhance chondrocyte biosynthesis by more accurately mimicking cartilage in the in vivo milieu. Phase I and Phase II were executed over a relatively condensed time frame (Day 16), while Phase III extended over a more extended duration (Day 21).

### 2.4 Cell Expansion, Encapsulation and Bioreactor Culture

Primary human chondrocytes were derived from a 22-year old male donor (P10970 Innoprot, Spain). Cells were expanded in T-75 culture flasks inside a chondrocyte basal medium (alpha minimum essential medium (α-MEM)), supplemented with 10% fetal bovine serum (FBS), 1% l-glutamine, 10 mM 4-(2-hydroxyethyl)-1-piperazineethanesulfonic acid (HEPES), 10 mM nonessential amino acids (NEEA), 1% penicillin, 1% streptomycin, 5 ng/mL fibroblast growth factor (FGF), and 1 ng/mL human recombinant transforming growth factor beta 1 (TGF-β1), up to passage 4.

Hydrogel precursor solutions were prepared, as described earlier, inside phosphate-buffered saline (PBS) containing LAP photoinitiator (lithium phenyl-2,4,6-trimethylbenzoylphosphinate, at a final concentration of 0.1 mg/mL). Passage 4 chondrocytes, at a seeding density of 10^7^ cells per mL, were resuspended in the hydrogel precursor and carefully pipetted into a custom-designed mold. The chondrocyte/hydrogel suspension was then cross-linked using a 405 nm wavelength light source for 2 min. The resulting constructs were cultured in a differentiation medium composed of FBS-free Dulbecco’s modified Eagle’s medium supplemented with insulin–transferrin–selenium (ITS-IV, 10%), l-ascorbic acid (VC, 1%), 10 ng/mL TGF-β1, and other additives (10 mM HEPES and 10 mM NEAA). The cell-seeded constructs were prepared in batches and subsequently randomly distributed among different study groups. Following the seeding step, all samples were precultured for 7 days in a cell growth medium within standard incubators (32.5 °C, 5% CO_2_, 21% O_2_). Next, the constructs were transferred to a bioreactor culture system where intermittent biomimetic thermomechanical signals (32.5–39 °C, ∼20% strain at 1 Hz every other day) and a low oxygen tension environment (4% O_2_) were applied until day 21. We replicated all forms of stimulation by utilizing a custom-made bioreactor that we developed in our laboratory.^14^ The compressive regime was specifically designed to imitate a normal physical activity. To model the temperature increase resulting from cyclic compression, we applied curve-fitting based on in vivo data during jogging over the same 1.5 h period and as previously described.^17^ After the stimulation was ceased, constructs were allowed to recover within standard incubators (32.5 °C, 5% CO_2_). Constructs were collected for analysis on days 16 and 21.

### 2.5 Quantitative Real-Time PCR

After 16 and 21 days of bioreactor culture, both stimulated and nonstimulated samples were promptly immersed in 0.3 mL of ice-cold TRIzol (Invitrogen) and stored at −80 °C for subsequent RNA isolation. To prepare the samples, TRIzol was added to each sample on ice, followed by vigorous homogenization. Subsequently, 0.1 mL of chloroform was added, and the mixture was hand-shaken for 15 s and then centrifuged at 4 °C for 10 min. The aqueous layer, containing RNA, was carefully collected and combined with an equal volume of 70% ethanol through pipetting. RNA isolation was performed using the Nucleospin XS kit according to the manufacturer’s instructions. The isolated RNA was quantified using a NanoDrop 1000 system and then reverse-transcribed into cDNA using the Taqman reverse transcription reagents (Applied Biosystems) in a 50 μL reaction volume, as previously described.^18^ The reaction mixture included the master mix, random hexamer, and the RNA sample.

For qRT-PCR analysis, Fast SYBR Green PCR Master Mix (Applied Biosystems) was employed in a final volume of 20 μL. Each reaction contained 1 μL of synthesized cDNA. The target genes selected for qRT-PCR included background potassium channel (TREK1), lysyl oxidase-like 2 (LOXL2), aggrecan (ACAN), sex-determining region Y-type (SRY, SOX9), piezo-type mechanosensitive ion channel component 1 (PIEZO1), transient receptor potential cation channel subfamily V member 4 (TRPV4), and collagen type II (COL2A). To ensure accurate normalization, ribosomal protein L13a (RPL13A) was utilized as the housekeeping gene. The relative gene expression of the experimental samples was determined using the ΔΔCT method. Primers were synthesized by Microsynth (Balgach, Switzerland).

### 2.6 Sample Preparation for Histology

To assess the production and distribution of the extracellular matrix, we employed histology. After 16 and 21 days, cell-hydrogel samples were harvested, and chondrocytes were fixed in 4% paraformaldehyde overnight at 4 °C. The following day, the samples were sequentially transferred to 15 and 30% sucrose solutions (Sigma) before being snap-frozen in an optimal cutting temperature compound (Sakura Tissue-Tek). Subsequently, the samples were sectioned into 7 μm thick slices using a standard cryostat. Imaging of histological slides from different samples was performed using the tile scan method with a 20× magnification on an Olympus VS120 whole slide scanner. Throughout the imaging process, the laser intensity and exposure duration were consistently set for all of the samples to ensure accurate comparisons.

### 2.7 Mechanical Characterization of Hydrogels

Cylindrical specimens with dimensions (diameter: 6 mm and height: 3 mm) were submerged in PBS and underwent unconfined compression experiments. The experiments were conducted using an Electropuls Dynamic Test System (Instron E3000, Instron, Norwood, Massachusetts, USA) at room temperature. The compressive loading was performed at a rate of 0.1 mm s^–1^, with the load–displacement data recorded. Swollen state samples underwent 30 compression cycles. The compressive modulus of the hydrogels was calculated through linear interpolation of the stress–strain curve during the last loading cycle, specifically between 12 and 15% strain (mm/mm). To determine the level of energy dissipation, the area enclosed by the hysteresis loop was measured and subsequently normalized to the volume of each sample.

### 2.8 Statistical Analysis

Statistical analysis was conducted using the analysis of variance (ANOVA), followed by Tukey’s post hoc tests for comparing mechanical data (*n* = 5) and gene expression data (*n* = 4) in multiple group comparisons. The results are presented as mean + standard deviation. The significance levels are denoted as (*) for *p* ≤ 0.05, (**) for *p* ≤ 0.01, and (***) for *p* ≤ 0.001. Statistical computations were performed by using Origin Pro 2021 software. Additionally, to validate gene expression patterns, two independent experiments were conducted, each of at least three technical replicates.

## 3 Results

### 3.1 Inclusion Complex Formation of Linear Polyethylene Glycol (PEG) and α-Cyclodextrin (α-CD)

**Figure 1:**
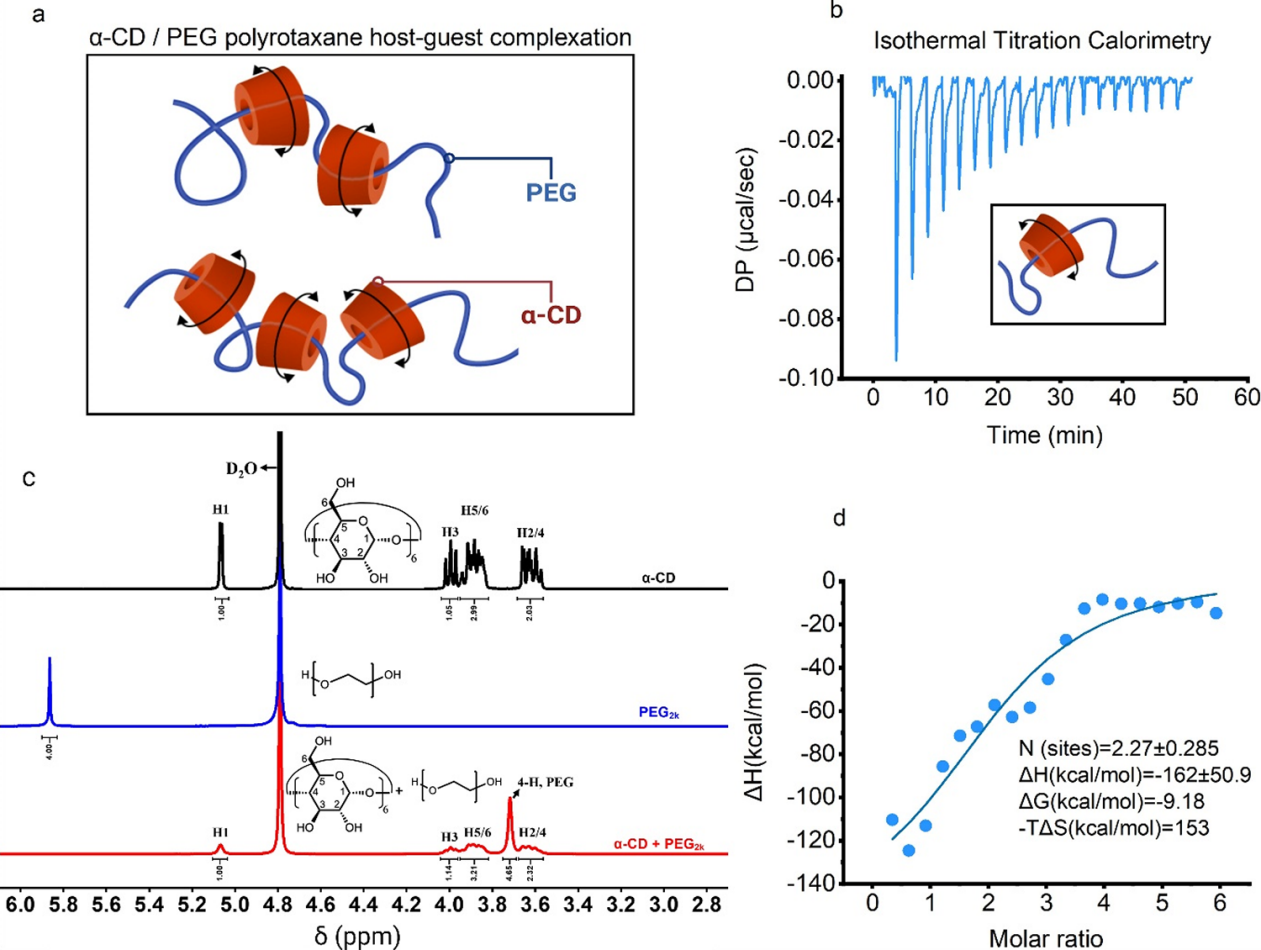
(a) Schematic illustration of elemental motion of polyrotaxanes. (b) ITC experimental curve for the titration of α-CD into PEG_2k_ solution at 298 K. It shows the heat evolved after each injection at the beginning and saturated after 12–13 injections. (c) Representative ^1^H NMR spectra of free α-CD (black line), free PEG_2k_ (blue line), and α-CD + PEG_2k_ complexes (red line). (d) Titration curve, which is obtained by the integration of the peaks from Figure 1b, together with a line of best fit, to estimate Δ*H*, Δ*G*, *T*Δ*S*, and the stoichiometry N.

Figure 1a shows a schematic illustration of typical polyrotaxanes consisting of a linear polymer chain (PEG) of 2 kDa molecular weight and threaded rings (α-CD). The confirmation and monitoring of inclusion complex formation were conducted through isothermal titration calorimetry (ITC). Each titration step and injection revealed distinct exothermic effects, as depicted in Figure 1b,d, presenting a characteristic ITC sigmoid thermogram. This behavior was attributed to a direct interaction between the PEG_2k_ chains and α-CD. The predominant negative enthalpy observed (Figure 1d, inset) suggested the involvement of a significant number of van der Waals interactions and host–guest complexations between PEG and alpha-CD, confirming a stoichiometric ratio of approximately 2.

It is important to note the inherent challenges in performing ITC between polymers and single molecules as multiple other interactions may occur, and chain–chain entanglement is likely to happen. However, upon analysis of the values, it indicates that two molecules of α-CD are complexed with a single polymer chain of PEG.

^1^H NMR spectroscopy was further utilized to investigate the relative position of the host–guest complexation between α-CD (a host) and the PEG chain (a guest). Figure 1c shows the NMR data of free α-CD (black line), free PEG (blue line), and α-CD + PEG complexes (red line). It is evident that upon incorporation into the host, the protons of the PEG_2k_ chain experienced a complete upfield shift (shielded), while the protons of the α-CD cage exhibited peak broadening compared to the free α-CD. This observation confirms the occurrence of host–guest complexation between α-CD and the PEG polymer chain.

### 3.2 Phase I: Dynamic Polyrotaxane Host–Guest Complexation Altered Gene Categories Encoding Multiple Signaling Pathways in Free-Swelling Hydrogels

Quantitative real-time PCR (qPCR) analysis was conducted on free-swelling hydrogels (labeled as 1–3) to discern the impact of host–guest complexation (polyrotaxanes) at the transcriptional level on day 16 of culture.

**Figure 2:**
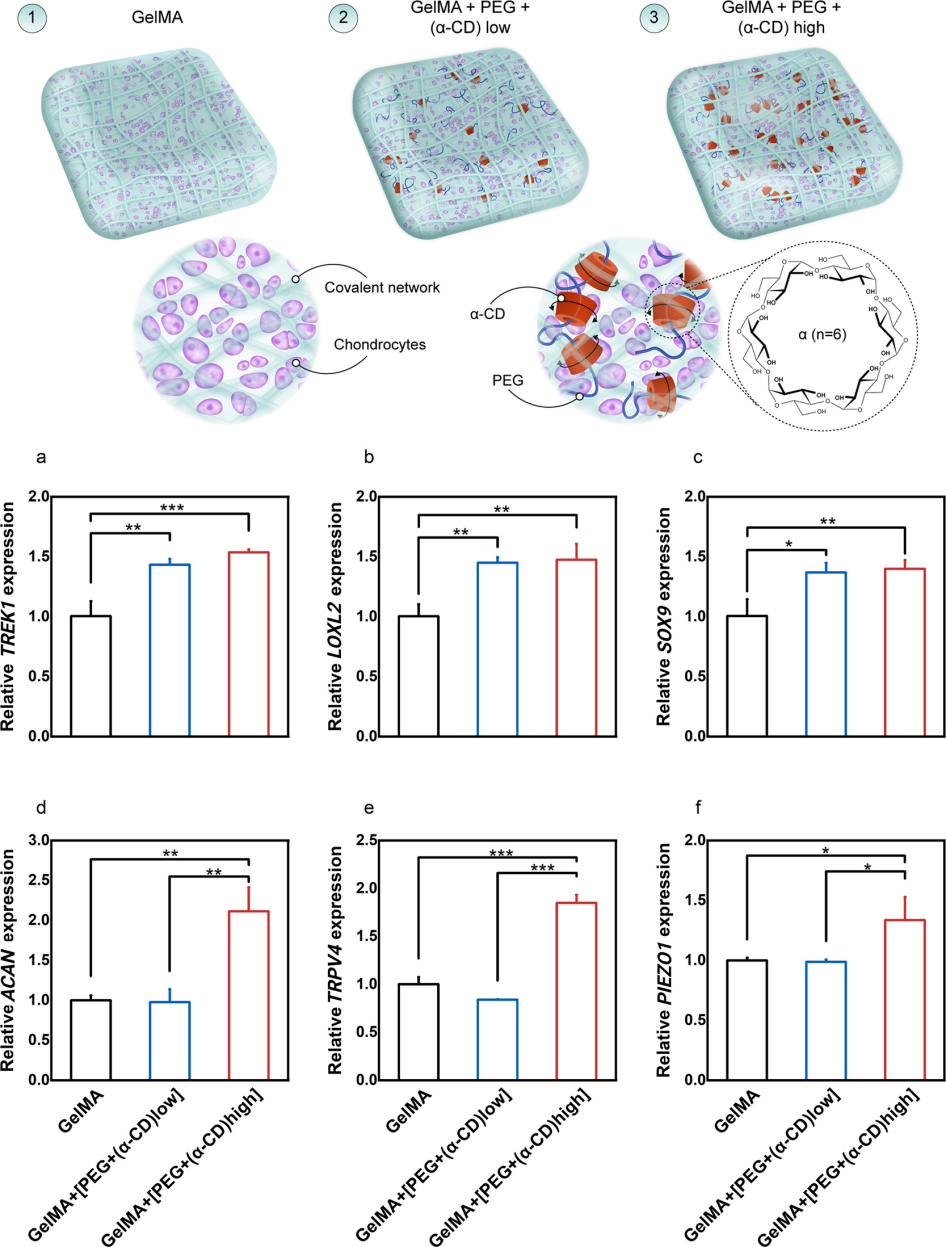
Schematic illustration depicting the various types of hydrogels employed in the study. The polyethylene glycol (PEG) concentration remained consistent across all supramolecular hydrogels, while the alpha-cyclodextrin (α-CD) concentration increased progressively from left to right (a–f). Comparison of the relative expressions of genes of interest, with RPL13a serving as the housekeeping gene.

The findings unveiled profound changes in the expression of genes implicated in crucial cellular signaling pathways (SRY-related HMG-box gene 9, SOX9), genes associated with matrix remodeling enzymes (Lysyl oxidase homologue 2, LOXL2), extracellular matrix proteins (including Aggrecan, ACAN), and thermomechano-regulated ion channels (including potassium and calcium transducers, TREK1, TRPV4, and PIEZO1), as illustrated in Figure 2a–f.

Overall, the inclusion of α-CD/PEG polyrotaxanes within the gelatin-based covalent network yielded mixed results worth noting. At lower concentrations, TREK1, LOXL2, and SOX9 genes displayed significant increases in expression, indicating a positive impact. However, transcription plateaued with higher concentrations, not contributing to further improvements in these genes (Figure 2a–c). Conversely, other major chondrogenic genes such as ACAN, PIEZO1, and TRPV4 exhibited no discernible changes at lower concentrations. Yet, an astonishing revelation emerged: these three genes demonstrated a synchronized response pattern, undergoing a remarkable increase in expression at higher concentrations spanning from 30 to 200% (Figure 2c–f).

These compelling results highlight the transformative influence of supramolecular host–guest complexation on the gene expression profile, underscoring the potential of this approach in modulating vital cellular pathways involved in chondrocyte physiology.

### 3.3 Phase II: Impact of Dynamic Host–Guest Polyrotaxanes on Sulfated Proteoglycans and Total Collagen Synthesis and Distribution during Free-Swelling Culture of Hydrogels

In cartilage tissue engineering, scaffolds must provide mechanical support while facilitating the deposition of extracellular matrix (ECM) by the embedded chondrocytes to promote the development of neocartilaginous tissue.^19,20^ In this series of experiments, we aimed to investigate the influence of double-network supramolecular hydrogels on the uniform secretion of the cartilaginous matrix during free-swelling conditions (without external stimulation up to day 16).

**Figure 3:**
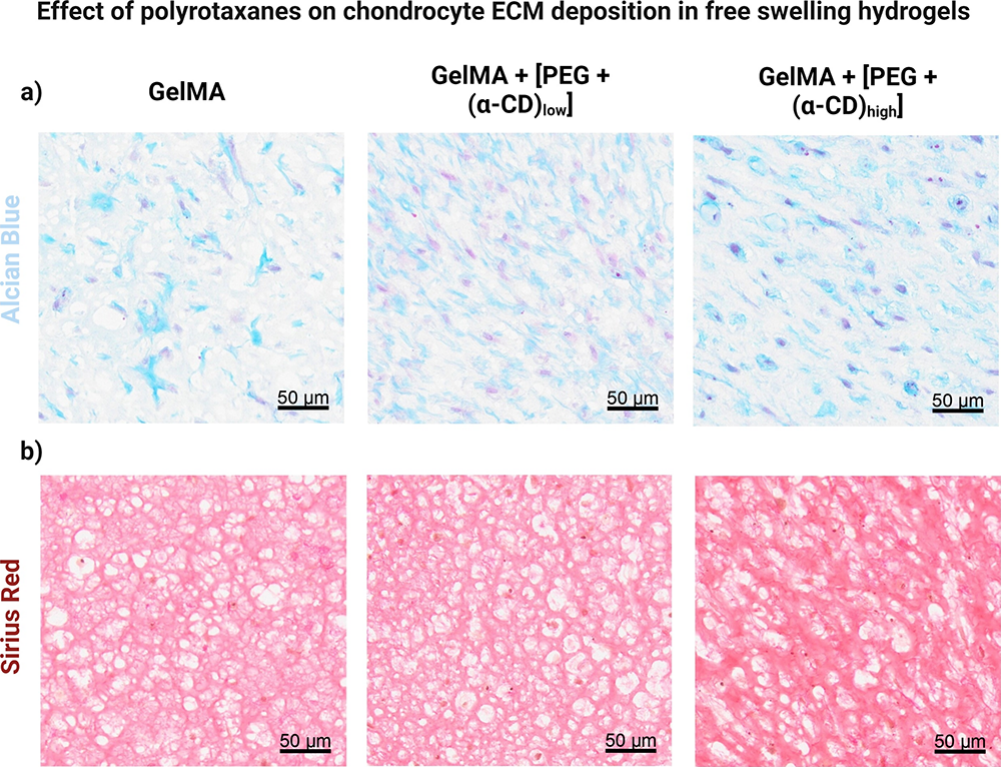
Histological analysis of neocartilage constructs under free-swelling conditions. (a) Representative images of Alcian Blue staining for sulfated glycosaminoglycan and glycoprotein content (blue). (b) Representative images of Sirius Red staining for general collagen content (red). The intensity of Alcian Blue staining is noticeably higher in supramolecular hydrogels with a higher concentration of alpha-cyclodextrin (α-CD) molecules. Additionally, Sirius Red staining reveals a localized increase as the concentration of α-CD molecules increases. Scale bar: 50 μm, objective 20×.

Collagens, as fibrillar proteins, play a crucial role in determining the shape and microarchitecture of articular cartilage.^21^ Sulfated glycosaminoglycans (sGAGs), the major components of proteoglycans such as aggrecan, contribute to water retention and provide compressive strength.^22^ We utilized histological staining techniques to examine the deposition of these key cartilage matrix molecules and assess the development of neocartilaginous tissues over time, as seen in Figure 3.

Consistent with the gene expression data, our results reveal significant variations in both collagen and sGAG contents, correlating with the concentration of α-CD/PEG polyrotaxanes within the hydrogels. Astonishingly, higher concentrations of α-CD which translate to higher dynamism in the hydrogel system (due to the higher level of α-CD/PEG complexations) have been discovered to foster a substantial accumulation of aggrecan, vividly exemplified by intensified Alcian Blue staining (Figure 3a). Furthermore, the diverse compositions of the hydrogels have illustrated distinct patterns in total collagen deposition with higher concentrations of α-CD showcasing an evident surge in the overall collagen accumulation, as depicted in Figure 3b.

### 3.4 Phase III: Supramolecular Host–Guest Complexation Modulates the Effects of Biomimetic Thermomechanical Stimulation on Chondrocyte Biosynthesis in a Dose-Dependent Manner

In this series of experiments, we extended the culture period from 16 to 21 days while applying biomimetic thermomechanical stimulation upon hypoxia treatment to the chondrocyte-laden hydrogels. Our results consistently demonstrate trends in both total collagen and glycosaminoglycan (GAG) content. Increasing concentrations of α-CD within the covalent-based network (while keeping the PEG concentration the same) demonstrated a notable capacity to promote augmented collagen accumulation (as shown in Sirius Red and Masson’s trichrome stainings), accompanied by significant deposition of sulfated GAGs (as shown in Alcian Blue and Safranin-O staining) compared to pure covalent-based hydrogels.

These outcomes underscore the pivotal role of host–guest supramolecular motifs in governing collagen and GAG secretion, thereby emphasizing the potential of double-network supramolecular hydrogels to facilitate the development of functional neocartilaginous tissue, especially upon biomimetic thermomechanical stimulation.

**Figure 4:**
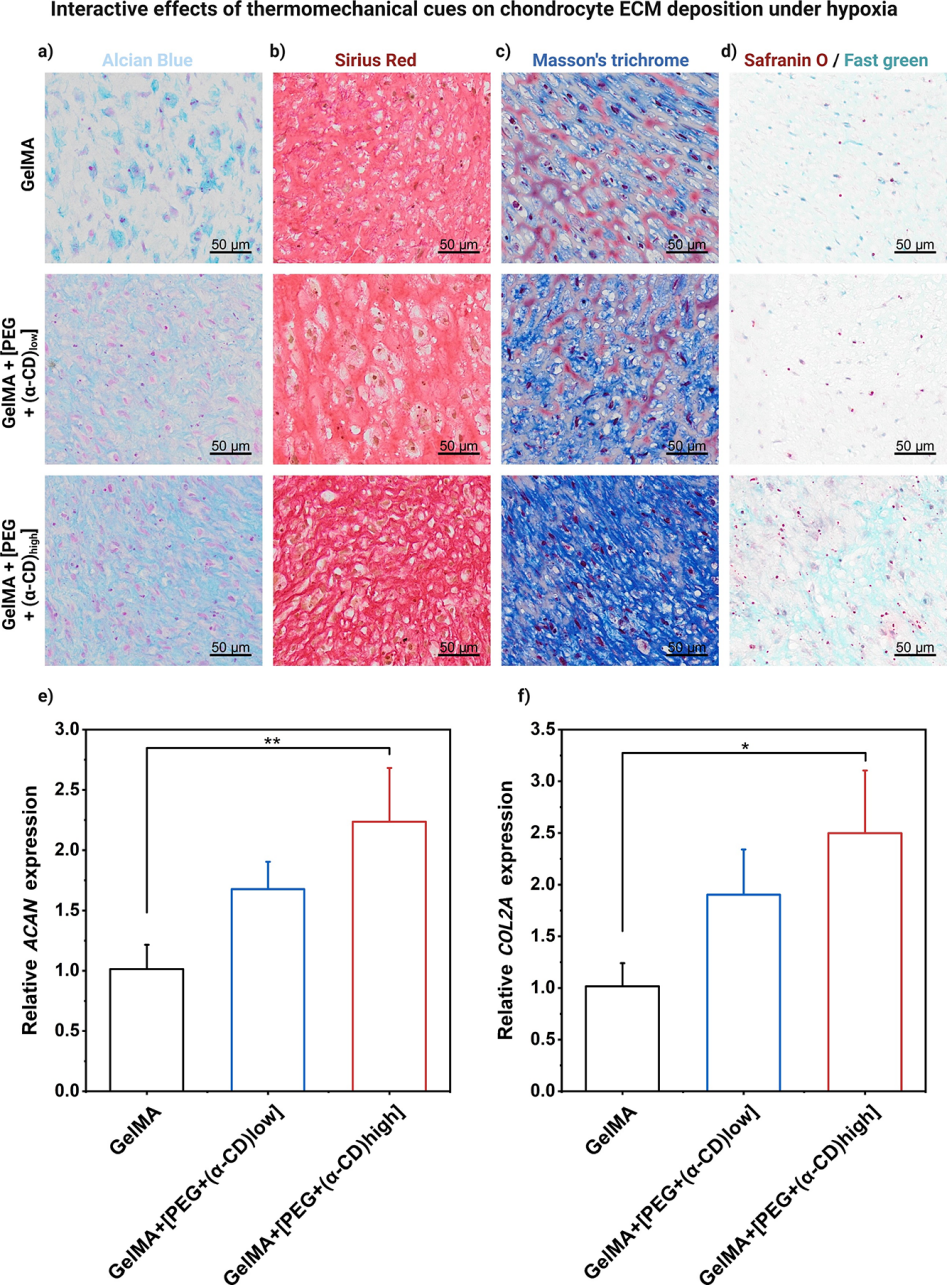
Interactive effects of mechanobiological cues on chondrocyte ECM deposition. (a) Representative images of Alcian Blue staining for sulfated glycosaminoglycan and glycoprotein content (blue). (b) Representative images of Sirius Red staining for general collagen content (red). (c) Representative images of Masson’s trichrome staining for the total collagen content (blue). (d) Representative images of Safranin-O/Fast green staining for sulfated glycosaminoglycan content are shown for the different types of hydrogels after the last loading cycle was ceased on day 21. Application of biomimetic thermomechanical loading under hypoxia significantly enhanced cartilage-related matrix accumulation, especially in the case of supramolecular hydrogels. **Alcian Blue**: sulfated GAGs and glycoproteins are stained blue, and the nuclei and cytoplasm pink, **Sirius Red**: collagen is stained red, and the nuclei dark brown, **Masson’s trichrome**: collagen is stained blue, and nuclei are stained dark brown, **Safranin-O/Fast green:** cartilage matrix will be stained orange to red, the nuclei will be stained black, and the background light green. Scale bar: 50 μm, objective 20×. (e) Comparison of the relative expressions of aggrecan (ACAN) and (f) collagen type II (COL2A) after the last loading cycle was ceased, with RPL13a serving as the housekeeping gene.

In addition to the histological staining, we analyzed the mRNA transcription of aggrecan (ACAN, Figure 4e) and collagen type II (COL2A, Figure 4f) genes among the different hydrogels. The intergroup comparisons aligned with the histological findings, demonstrating a substantial upregulation of these pivotal genes, with an increase of 200–250%, respectively.

## 4 Discussion

The interdependence of chondrocytes and their extracellular matrix is a fundamental aspect of cell behavior and function.^23^ To comprehend these relationships in vitro, hydrogels are a promising candidate due to the possibility of precisely controlling their chemical and physical properties. However, existing studies have predominantly utilized purely covalently cross-linked hydrogels, which fail to confer the essential dynamicity found in the ECM of native tissues.^24,25^ Supramolecular hydrogels offer a dynamic noncovalent alternative that more accurately reflects the native environment of hyaline cartilage.^26^ Utilizing supramolecular noncovalent motifs such as polyrotaxanes, this study aimed to engineer double-network hybrid hydrogels with the ability to reorganize under free-swelling conditions. More specifically, we have developed a method that integrates a covalent-based hydrogel with supramolecular polyrotaxane patterns with the intention of harnessing the advantageous attributes of supramolecular host–guest interactions (characterized by dynamic reversibility) within the covalent network. The addition of supramolecular polyrotaxanes into the covalent network hydrogels was intended to enhance the biosynthetic capacity of the neocartilage constructs. The underlying hypothesis suggested that by replicating the natural characteristics observed in the cartilage extracellular matrix (e.g., inherent dynamic network of molecules), chondrocytes would be prompted to produce essential matrix proteins. Subsequently, the investigation delves into the biophysical impacts of applied thermomechanical loads on chondrocytes encapsulated in these double-network hydrogels. Given that native cartilage experiences transient thermal cues during joint loading upon a hypoxic environment, replicating these interactions in vitro became a focal point for assessing the potential acceleration of chondrocyte biosynthesis. The thermomechanical stimulation protocol used was similar to that used in our previous investigations.^14,27^

Through our rigorous investigation, we have uncovered a fascinating interplay between double-network supramolecular hydrogels, thermomechanical loading, and hypoxic conditions in regulating chondrogenesis. Our research unveiled that when polyrotaxanes are incorporated into the covalent network, especially at increased concentrations, alongside thermomechanical loading and reduced oxygen tension, a significant increase in chondrogenic markers and cartilage-related proteins is observed. This synergistic effect suggests a unique interplay between these cues that holds significant promise in the field of cartilage tissue engineering, highlighting the importance of a multifaceted approach to this complex challenge.

Remarkably, our findings indicate a substantial augmentation in chondrogenesis within the hybrid supramolecular hydrogels, surpassing that observed in the covalently cross-linked (single network) hydrogel counterparts. Moreover, a discernible relationship emerges, whereby the extent of host–guest complexation (polyrotaxanes) exhibits a direct correlation with the magnitude of the chondrogenic differentiation. This positive correlation is substantiated through quantifiable variations in mRNA expression levels of critical chondrogenic marker genes, namely, SOX9, ACAN, and LOXL2. Such improvements were detected at the protein level as well. These findings are consistent with previous research^27,7,28^ supporting the notion that hydrogels incorporating supramolecular components, particularly those featuring stronger host–guest interactions, offer a promising platform for the development of tissue-engineered constructs with superior chondrogenic potential.

In our investigation, the hydrogel formulations were also subjected to bioreactor culture conditions that incorporated thermomechanical signals under a low oxygen tension. Our previous studies have extensively elucidated the significance and relevance in replicating cartilage self-heating in vitro and demonstrated thoroughly how loading-induced evolved temperature can be harnessed in vitro to accelerate tissue maturation through expression of major structural proteins.^12,14,29^ Building upon our prior findings where load, heat, and hypoxia interactions were studied, herein we aimed to introduce the inherent noncovalent nature of the culture environment as an additional parameter in this multifaceted equation. The relevance of including PEG/alpha cyclodextrin-based polyrotaxanes into the robust covalent network stems from their capacity to partially replicate the dynamic interactions found in natural cartilage.^30,31^ Through the incorporation of supramolecular polyrataxanes, we aim to mimic the innate noncovalent interactions inherent in cartilage tissue, playing a pivotal role in its mechanical composure and potential signals they transfer to chondrocytes. Our analysis of the resulting protein synthesis levels in the stimulated samples revealed that the double network hydrogels exhibited a notable upregulation of cartilage matrix proteins compared to the pure covalently cross-linked hydrogel.

Our findings strongly support the notion of the pivotal role of ion channels in mediating the effects of thermomechanical loading on chondrocytes. We identified three ion channels, namely, TREK1, TRPV4, and PIEZO1, whose expression was significantly upregulated in the presence of host–guest reversible polyrotaxanes. While it could be argued that our channel analysis has only been performed in free-swelling constructs and not in stimulated hydrogels, we have previously shown that thermomechanical loading increases the expression of these channels at protein levels as well.^14,29^ Hence, we contend with the validity of our hypothesis, suggesting that the observed effects likely arise from the interplay between various stimuli and the multifaceted responses of chondrocytes.

We also conducted a systematic investigation into the distinct influences stemming from the separate incorporation of PEG and alpha cyclodextrin moieties into the covalent network. Interestingly, we found no significant changes in the expression of major genes among the different experimental groups, indicating that the observed effect primarily arises from the complexation of alpha cyclodextrin and PEG (see Figure S1). To ensure the validity of our observations, we diligently measured the bulk mechanical properties of the hydrogels, specifically focusing on energy dissipation levels and hydrogel stiffness before cell encapsulation (see Figure S2). Encouragingly, our results demonstrated consistent mechanical attributes across the experimental groups (prior to cell encapsulation), suggesting that no other parameter significantly influenced the observed effects.

The rheological properties of the hydrogel were systematically analyzed. The results indicate no significant difference in the storage modulus (*G*′) between GelMa and GelMa incorporating high concentration of host–guest polyrotaxanes, as depicted in Figure S3. Despite subtle variations in rheological characteristics, our focus lies on the consequential biological implications. Even at these lower concentrations, the presence of host–guest molecules significantly influences the chondrocyte response. These findings lend strong support to the relevance and reliability of our conclusions.

## 5 Conclusions

In conclusion, this study provides crucial insights into the dynamic nature of cartilage, even under free-swelling conditions, and the role of dynamic polyrotaxanes, thermomechanical loading, and hypoxia in regulating chondrogenesis. By incorporating supramolecular motifs into a covalent-based hydrogel system, we aimed to partially mimic the intricate dynamic and noncovalent interactions present in natural cartilage. Supramolecular hydrogels with high amount of host–guest cross-links, when subjected to thermomechanical stimulation and hypoxic conditions, enhance chondrogenic markers and proteins. This highlights their potential as a promising platform for advanced tissue-engineered constructs with improved chondrogenic capabilities.

## Data Availability

The data sets generated and/or analyzed during the current study are available from the corresponding author on reasonable request.

## Supporting Information Available

Additional experimental details including gene expression comparisons in free-swelling controls and comparisons of mechanical and rheological properties of the hydrogels (PDF)
