# Supplementary material for: Impact of Molecular Dynamics of Polyrotaxanes on Chondrocytes in Double-Network Supramolecular Hydrogels under Physiological Thermomechanical Stimulation
Source: Biomacromolecules. 2024 Jan 2;25(2):1144–52. doi: 10.1021/acs.biomac.3c01132 (PMC10865359; doi:10.1021/acs.biomac.3c01132)
Supplement: Supplementary file 1 — bm3c01132_si_001.pdf [file bm3c01132_si_001.pdf]

Impact of Molecular Dynamics of Polyrotaxanes on Chondrocytes in Double Network Supramolecular Hydrogels under Physiological Thermomechanical Stimulation

Theofanis Stampoultzis[1†], Vijay Kumar Rana [1†], Yanheng Guo[1†], Dominique P. Pioletti[1*]

[1]Laboratory of Biomechanical Orthopedics, Institute of Bioengineering, EPFL, Switzerland

† Equal contribution

[*]Correspondence to: Dominique P. Pioletti

Author information:

1. Theofanis Stampoultzis

Email: theofanis.stampoultzis@epfl.ch

2. Vijay Kumar Rana

Email: vijay.rana@epfl.ch

3. Yanheng Guo

Email: yanheng.guo@epfl.ch

4. Dominique P. Pioletti

Phone: +41 21 693 83 41

Email: dominique.pioletti@epfl.ch

Effect of pure PEG and pure α-CD on gene expression

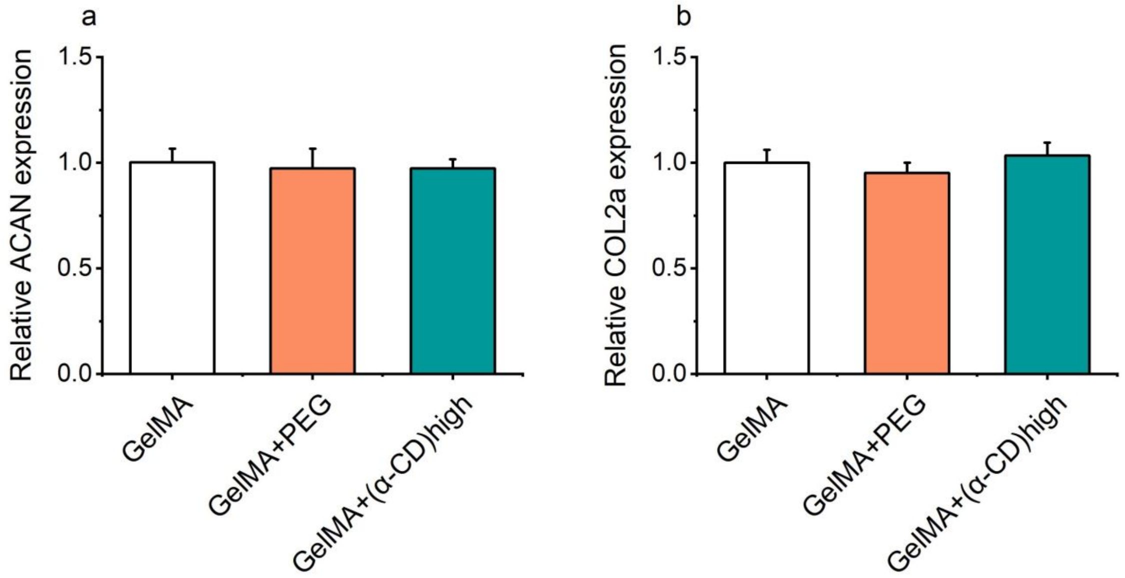

Figure S1 – Relative gene expression of Aggrecan and Collagen type II did not result in significant changes due to single PEG or α-CD molecules.

Differences in Mechanical characteristics of different hydrogels

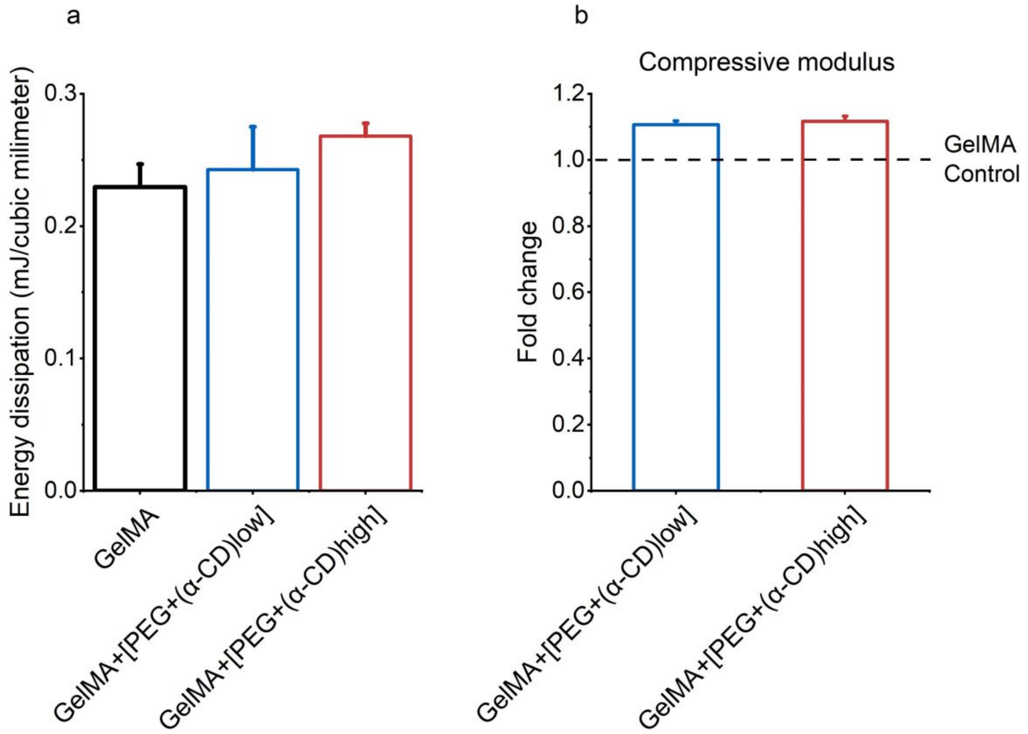

Figure S2 – Energy dissipation level of different hydrogels and fold change of hydrogel stiffness with respect to host-guest incorporation within the GelMA network. No significant changes were observed.

Differences in Rheological properties due to incorporation of polyrotaxanes

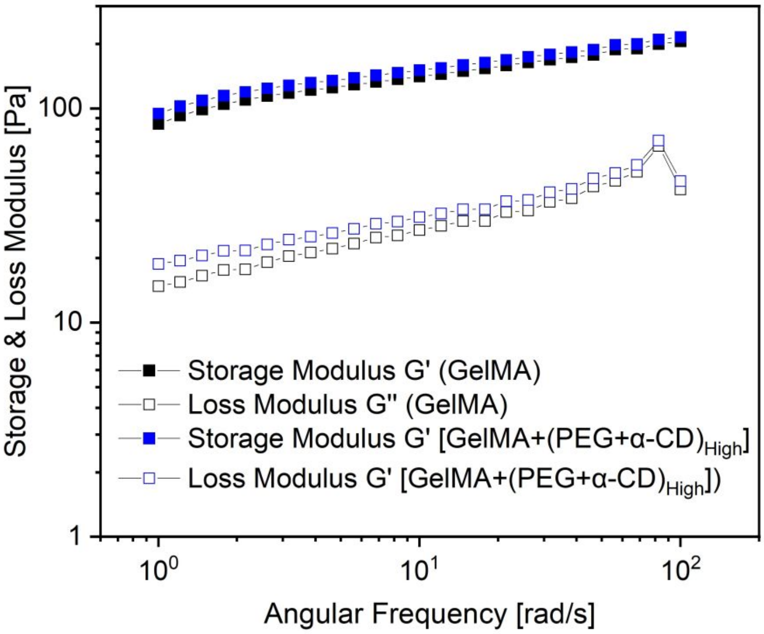

Figure S3 - Oscillatory rheological data: representative frequency sweeps to analyze the shear modulus of the hydrogels. Data presented here correspond to an average of 4 samples. We compared only the pure covalent network and the supramolecular hydrogels with the highest concentration of cyclodextrin molecules.
